# Breakfast consumption trends among young Australian children aged up to 5 years: results from InFANT program

**DOI:** 10.3389/fendo.2023.1154844

**Published:** 2023-08-10

**Authors:** Seon Y. Park, Penelope Love, Miaobing Zheng, Karen J. Campbell, Kathleen E. Lacy

**Affiliations:** Institute for Physical Activity and Nutrition (IPAN), School of Exercise and Nutrition Sciences (SENS), Deakin University, Geelong, VIC, Australia

**Keywords:** breakfast, early childhood nutrition, breakfast consumption trends, food group intakes, nutrient intakes

## Abstract

Breakfast is considered a healthy dietary habit which can track over time from childhood to adulthood. The breakfast meal has the potential to improve daily dietary quality, particularly if it includes a range of food groups and adequate nutrient intakes. However, research on breakfast consumption trends among young Australian children aged up to 5 years is currently limited. This study assessed children’s usual breakfast food group and nutrient intakes at ages 1.5 (n = 369), 3.5 (n = 242), and 5.0 (n =240) years using three 24-hour dietary recalls from the Melbourne InFANT program. Tracking of food groups at breakfast across the three ages was assessed by Pearson correlation of energy-adjusted food intake residuals. The main food groups consumed at breakfast were grains, milk/alternatives and discretionary items, with vegetables rarely consumed at any age. Our study found that while breakfast contributed about 20% of total daily energy, this provided 20%-29.1% of total daily intake across all ages for carbohydrates, total sugars, calcium and potassium. For the contribution to daily recommendations, breakfast contributed more than about a third of daily recommended intakes for some micronutrients (e.g., iron, calcium and zinc), and a large proportion (over 40%) of sodium intake. Children consumed 11.9% -15.2% of their energy at breakfast from saturated fat, which is higher than the recommended total energy contribution of saturated fat (no more than 10% from saturated fat). For tracking of most food groups and nutrients, tracking was found to be low or moderate over time. Given the contribution that breakfast can make to ensure children achieve their daily dietary intakes, early interventions for young Australian children should focus on practical strategies to increase vegetable intake while reducing sodium and saturated fat intake at breakfast.

## 1 Introduction

Early childhood, broadly defined as from birth to five years of age, is an important time to provide the basis for lifelong health related to nutrition (1). It is a period of relatively fast growth with changes in physiological and nutritional needs, and exposure to healthy eating in this period is likely to have a long-term impact (2). Furthermore, this is when taste and food preferences are developed, and the eating habits formed during this period are expected to continue into adulthood (3). As such, the early establishment of healthy eating habits in young children is important.

Breakfast consumption is considered a long-term healthy dietary habit to be established from early childhood (4), contributing to overall diet quality in terms of total daily energy, nutrient and food group intakes (5–7). Several studies have reported that both children and adolescents aged between 2 and 18 years, who consumed breakfast, had higher daily intakes of fruits and vegetables than those who did not consume breakfast (8–11). Breakfast consumers were also more likely to meet Estimated Average Requirements (EAR) for calcium and iron, which play vital roles in children’s health (8, 12, 13). Given that breakfast plays an essential role in improving overall dietary quality for children, knowing what food groups and nutrients are consumed at breakfast during early childhood and tracking these intakes over time can inform critical starting points for intervention to promote optimal nutrition from early life.

Most studies on breakfast have had cross-sectional designs and primarily focused on school-aged children and adolescents (14–16). Much of the existing research on breakfast in young people has been reported through the International Breakfast Research Initiative (IBRI) using nationally representative dietary surveys from Canada (5), Denmark (17), France (18), Spain (19), United Kingdom (6), United States (20), and Japan (21). Similarly, studies in Australia have investigated breakfast consumption among children and/or adolescents aged 2-18 years (8, 11, 12) using nationally representative cross-sectional dietary surveys. While these cross-sectional studies have provided important information on foods and nutrients consumed at breakfast, they do not provide insights on longitudinal changes in foods and nutrients consumed at breakfast. Therefore, how breakfast intakes change throughout early childhood remains unclear.

Longitudinal studies on breakfast consumption in childhood are limited and have not focused specifically on food and nutrient intake changes across early childhood. One study conducted in Germany tracked breakfast consumption across childhood and into adolescence between 1986 and 2007, reporting that 2- to 18-year-old German children showed a decline in breakfast consumption with age (22). Of note, this study reported breakfast intakes for children aged 2-5 years combined rather than reporting by individual ages across this period. One study in Australia reported longitudinal results on breakfast skipping for children aged 2-5 years, reporting that boys whose mothers were obese when they were 2-3 years of age were more likely to skip breakfast when they were 4-5 years of age. This study, however, did not report on nutrient and food group intakes (23).

To our knowledge, previous research on breakfast consumption in childhood has mainly focused on children who were over 2 years of age and/or only presented results on children 2-5 years as one group, primarily used cross-sectional study designs, and often focused on behaviours such as breakfast skipping. Also, previous Australian studies have shown that a relatively high proportion (over 80%) of young Australian children aged 2 to 5 years consume breakfast (24, 25), but no study has described food group and nutrient consumption at breakfast and assessed longitudinal tracking of consumption across early childhood in young Australian children. Therefore, the aims of this study were (i) to describe the mean breakfast intakes of food groups and nutrients for children at ages 1.5, 3.5 and 5 years, and (ii) to assess tracking of breakfast food groups and nutrient intakes across the first five years of childhood.

## 2 Methods

### 2.1 Study design and participants

This study is a secondary analysis utilising data from the Melbourne Infant Feeding, Activity and Nutrition Trial (InFANT) Program (2008-2010) which was a 15-month cluster-randomised controlled trial aimed to prevent childhood obesity in Melbourne, Australia (26). The InFANT Program recruited 542 participant pairs (child-parent pairs with child 4 months of age at baseline) from 62 existing first-time parent’s groups to deliver a healthy feeding, diet and physical activity intervention targeting infants aged 4-18 months. All participants provided informed consent. In the InFANT study, demographic data were collected at two time points when children were approximately 9 and 20 months of age (26). Dietary data were collected at age 1.5 years at intervention conclusion (27) and at two follow-ups with no intervention (2011-2013) when children were aged 3.5 and 5.0 years (28). The InFANT study included first-time parents who could communicate in English. Parents who could not communicate in English and/or had children with chronic diseases were excluded (26). The InFANT study has ethical approval from the Deakin University Ethics committee (ID number: EC 175-2007) and the Victorian Office for Children (Ref: CDF/07/1138).

For the current analysis, we added in extra inclusion and exclusion criteria. Children were included in the present study if their parent provided demographic information and dietary intake data when they were 1.5, 3.5 and 5 years. We excluded children if they did not consume breakfast; or their mothers were not first-time parents; or they had less than three 24-hour dietary recalls at a data collection time; or they were considered outliers for energy intakes according to the criterion of mean ±3 SD (29). We conducted preliminary tests to check for differences in the breakfast data between control and intervention groups. As breakfast data analysed in the present study did not show statistically significant differences in food items or nutrient intakes, data from the intervention and control groups were pooled to maximise the sample size in this study.

### 2.2 Assessment of dietary intake

The InFANT study involved a wide range of professional and academic staff with skills across nutrition, dietetics, psychology, physical activity and maternal and child health (26–28).

Children’s dietary intakes were assessed by trained nutritionists *via* parent report when children were 1.5, 3.5 and 5 years of age. Parents were asked to recall all food and beverages consumed by their child during the previous 24 hours using a telephone-administered 5-pass recall procedure (26), based on methods validated by the U.S. Department of Agriculture and used in the U.S. Feeding Infants and Toddlers Study (30). Recalls were collected on three non-consecutive days, including two weekdays and one weekend day. A food measurement booklet was provided to parents to help them with portion size estimation. The collected 24hr recall data were coded into food and nutrient intakes using the 2007 Australian Food and Nutrient database (AUSNUT) (31). Each item was coded with an 8-digit AUSNUT code. In cases where an 8-digit AUSNUT code did not exist for a food item consumed by children, items were assigned purpose-designed InFANT 8-digit food codes and relevant nutrition information was derived from product manufacturers. Dietitians checked the dietary data for accuracy following coding (32). After dietary data collection, entry, and checking, the nutritional analysis was usually done by one nutritionist/dietitian (26).

In the present study, all foods and beverages (other than water) consumed were classified into nine major food groups based on the 2013 Australian Dietary Guidelines (ADGs) using the AUSNUT 2007 5-digit food codes (31): grains, wholegrains, milk/alternatives, meat/alternatives, fruits, vegetables, discretionary foods, infant cereal products and infant formulae (33) (**Supplementary Table 1**). These nine major food groups were further categorised into sub-major food groups using AUSNUT 2007 8-digit food codes and purpose-designed InFANT 8-digit food codes (31) to enable examination of the top two or three food sources within each major food group. Since the ADGs recommend that most grain foods should be consumed as wholegrain foods (33), a subgroup of wholegrain foods was included in this study (34). As per wholegrain ingredient content claims that only a product with wholegrain ≥8g is allowed to be declared wholegrain (35), we applied a stricter wholegrain definition using 8-digit AUSNUT food codes and InFANT 8-digit food codes. Discretionary foods were defined as energy-dense and nutrient-poor foods according to the criteria described in the Australian Bureau of Statistics discretionary flag list using 8-digit AUSNUT codes (36). Infant foods and formulae/breastmilk were classified into their own food groups to help identify the transition from greater to lesser consumption of these foods and beverages across early childhood (31).

Energy and selected nutrient intakes at breakfast were derived using AUSNUT 2007 (31) and compared with the Nutrient Reference Values for Australia and New Zealand (NRVs) (37). The macronutrients investigated were protein, total and saturated fat, total sugars, carbohydrates and fibre. The micronutrients investigated were iron, calcium, vitamin C, folate, potassium, sodium and zinc. These nutrients were selected based on previous research from the International Breakfast Research Initiative and known to play important roles in early childhood development (6, 21, 38). To assess nutrient adequacy, the Estimated Average Requirement (EAR) was used and Adequate Intake (AI) was used when EAR was not available (37).

### 2.3 Assessment of breakfast intake

As there is no single definition of the breakfast meal used consistently in the literature, initial exploration was conducted to identify breakfast commencement, conclusion and duration for young children. In this study, breakfast was initially defined as the first eating and/or drinking occasion occurring between 5am and 10am. Meal occasion durations of 15, 30 and 60 minutes were considered as these three eating durations are widely used in the literature (39, 40). However, the 15- and 30- minute durations were considered inappropriate for use in the present study as they often failed to capture many breakfast foods and/or included water only. About 45% of children in the sample were found to consume breakfast-type foods beyond 30 minutes, and an eating duration of 60 minutes captured most breakfast food and beverage intakes. As a result, breakfast was defined as all foods and drinks consumed within 60 minutes of the first eating or drinking occasion occurring between 5am and 10am.

### 2.4 Statistical analysis

Usual breakfast intakes across three non-consecutive days were estimated at each age for food groups (g/day), macronutrients (g/day), micronutrients (mg; micrograms/day or ug DFE) and energy consumption (kJ/day) using the Multiple Source Method (MSM) (41). For each major food group (grains; milk/alternatives; fruits; vegetables; meat/alternatives), wholegrains, discretionary foods, infant cereals, and infant formulae), the proportion (%) of children consuming the food (yes (coded 1)/no (coded 0)) was assessed. The top two or three sub-major food items consumed at breakfast were determined and ranked by the prevalence of consumers (42).

When assessing tracking of breakfast intakes of food groups and selected nutrients, food groups and nutrients were adjusted for age, biological sex at birth, and total energy intakes for the day using the residual method. Pearson partial correlation coefficients were calculated between adjusted food group intakes at 1.5, 3.5, and 5.0 years. Correlation coefficients were defined as: low < 0.3; moderate 0.3–0.6; and high > 0.6 tracking/correlation (43, 44). All analyses were conducted using Stata software (Release 16.0; StataCorp LP, College Station, TX, USA), and all p-values <0.05 were considered statistically significant.

## 3 Results

### 3.1 Sample size and characteristics

At each time point, n=428 children aged 1.5 years, n=261 children aged 3.0 years, and n=270 children aged 5 years were followed up from n=542 children at baseline in the InFANT study. Among them, several children were excluded from data analyses because they did not consume breakfast (excluded n=2 at age 3.5 years); or their mothers were not first-time parents (excluded n=12 at age 1.5 years, n=9 at age 5.0 years); or they had less than three 24-hour dietary recalls at a data collection time-point (excluded n=44 at age 1.5 years, n=5 at age 3.5 years, n=20 at age 5.0 years); or they were considered outliers for energy intakes according to the criterion of mean ±3 SD (excluded n=3 at age 1.5 years, n=10 at age 3.5 years, n=1 at age 5.0 years). These exclusions resulted in the sample of children being n=369; n=244 and n=240 for ages 1.5, 3, and 5 years, respectively (**Supplementary Figure 1**).

Sample characteristics are shown in **Table 1**. Most child and maternal characteristics were similar across the three ages. There was a small variation in the proportion of male and female child participants across the time points (54% males and 46% females participated at 1.5 years, 50% males and females participated at 3.5 years, and 52% males and 48% females participated at 5.0 years). At all ages, children were most likely to come from families with relatively highly educated, Australian-born mothers.

**Table 1:** Demographic characteristics of children aged 1.5, 3.5 and 5.0 years in the Melbourne Infant Feeding Activity and Nutrition Trial (InFANT) Program.

Characteristics	1.5 years	3.5 years	5.0 years
Child Participants (n)	369	244	240
	Mean ± SD	Mean ± SD	Mean ± SD
Child			
Male (%)	54.0	50.0	52.0
Female (%)	46.0	50.0	48.0
Birth weight (kg)	3.4 ± 0.6	3.4 ± 0.6	3.4 ± 0.6
Gestational age (weeks)	39.2 ± 2.4	39.1 ± 2.6	39.1 ± 2.4
Breastfeeding duration (months)	7.6 ± 5.0	8.0 ± 5.1	8.1 ± 5.0
BMI-z score ^a^	0.8 ± 1.0	0.7 ± 0.8	0.6 ± 1.0
Mother			
Age at baseline (years)	32.3 ± 4.3	32.5 ± 4.0	32.4 ± 3.9
Education level (%)			
Low (High school)	19.5	17.2	17.1
Intermediate (trade and certificate qualifications)	21.4	19.3	20.0
High (University degree)	59.1	63.5	62.9
Country of birth (%)			
Australia	79.1	82.8	81.3
Other	20.9	17.2	18.7
Language spoken at home (%)			
English	94.0	95.5	95.0
Other	6.0	4.5	5.0

a Body Mass Index (BMI)- z score was calculated as age-adjusted zBMI based on WHO cut-offs.

### 3.2 Food group and nutrient intakes at breakfast

Food group and nutrient intakes of children from the intervention and the control groups at ages 1.5, 3.5 and 5 years were similar. They did not show statistically significant differences between control and intervention groups (**Supplementary Tables 2, 3**).

#### 3.2.1 Food group intakes at breakfast

The mean intakes of food groups consumed at breakfast across each time point are shown in **Table 2**. Across the three ages, the most frequently consumed food group at breakfast was grains (81.0%-94.6% of children), followed by milk/alternatives (74.6%-79.5% of children), wholegrains (30.8%-64.5% of children), and discretionary foods (24.4%-48.3% of children). Few children consumed meat/alternatives (4.9%-14.2% of children) or vegetables (0.5%-2.9% of children). In terms of the amount of food consumed, major food groups consumed at breakfast were milk/alternatives (140.0g-146.0g) and grains (38.0g-59.0g), followed by discretionary foods (34.0kJ-92.0kJ), then fruits (23.0g-32.0g) and wholegrains (13.0g-25.0g). The least consumed food groups at breakfast were meat/alternatives (≤ 4.5 g) and vegetables (≤1.4g) across all ages.

**Table 2:** Usual intakes per capita and consumers (%) of food groups at breakfast (g/three non-consecutive days) calculated by Multiple Source Method (MSM) of children aged 1.5, 3.5 and 5.0 years in the Melbourne Infant Feeding Activity and Nutrition Trial (InFANT) Program.

Food group (g)	1.5 years (n=369)		3.5 years (n=244)		5.0 years (n=240)	
	Consumers	Intakes	Consumers	Intakes	Consumers	Intakes
	(%)	Mean (SD)	(%)	Mean (SD)	(%)	Mean (SD)
Grains	81.0 - 81.2	38.0 (31.0)	87.7 - 89.3	53.0 (39.0)	91.7 - 94.6	59.0 (36.0)
Wholegrains ^a^	59.3 - 64.5	25.0 (23.0)	59.4 - 63.5	22.0 (21.0)	30.8 – 42.0	13.0 (18.0)
Milk/alternatives	78.0 -79.1	140.0 (93.0)	77.9 - 79.5	146.0 (86.0)	74.6 - 76.3	141.0 (82.0)
Meat/alternatives	4.9 - 6.2	2.4 (7.4)	11.1 - 11.9	3.4 (7.9)	11.3 - 14.2	4.5 (12.0)
Fruit	31.2 - 38.2	23.0 (29.0)	31.5 - 34.4	32.0 (42.0)	29.6 - 30.8	31.0 (43.0)
Vegetable	0.5 - 2.4	0.5 (2.5)	0.8 - 2.9	0.5 (2.2)	1.3 - 2.5	1.4 (8.0)
Discretionary food (kJ)	29.5 - 32.8	34.0 (52.0)	24.4 – 39.8	72.0 (89.0)	46.3 - 48.3	92.0 (98.0)
Infant cereal	1.8 - 3.8	0.8 (5.7)	0.0	0.0 (0.0)	0.0	0.0 (0.0)
Infant formula	11.7 - 12.7	16.0 (44.0)	0.4 – 1.2	2.6 (22.0)	0.8 – 1.3	1.6 (14.3)

a wholegrain is a sub-item as part of grains.

Consumers (%) were defined as ‘consuming (yes/no) of each food group’ so consumers (%) represent the range for % of total children consuming each food group over three 24-hour dietary recalls.

The proportions of children consuming common food sources within the nine major food groups are shown in **Supplementary Table 4**. Across all three ages, breakfast cereal was the most frequently consumed grain item at breakfast, followed by bread and bread rolls. The most frequently consumed wholegrains (mixed grain cereal and wholemeal bread) were the same across ages, with the proportion of mixed grain cereal consumers decreasing over time. With respect to discretionary food items, vegemite (yeast extract spread) was the most frequently consumed item by children aged 1.5 years, while honey, sugar, and concentrated sugar-based syrup became the main discretionary item consumed by children aged 3.5 years and 5.0 years. Within the vegetable food group, avocado was the most frequently consumed vegetable at breakfast (< 3.0% of children) across the three ages.

#### 3.2.2 Nutrient intakes at breakfast

##### 3.2.2.1 Contribution (%) to total daily intakes

**Figure 1:**
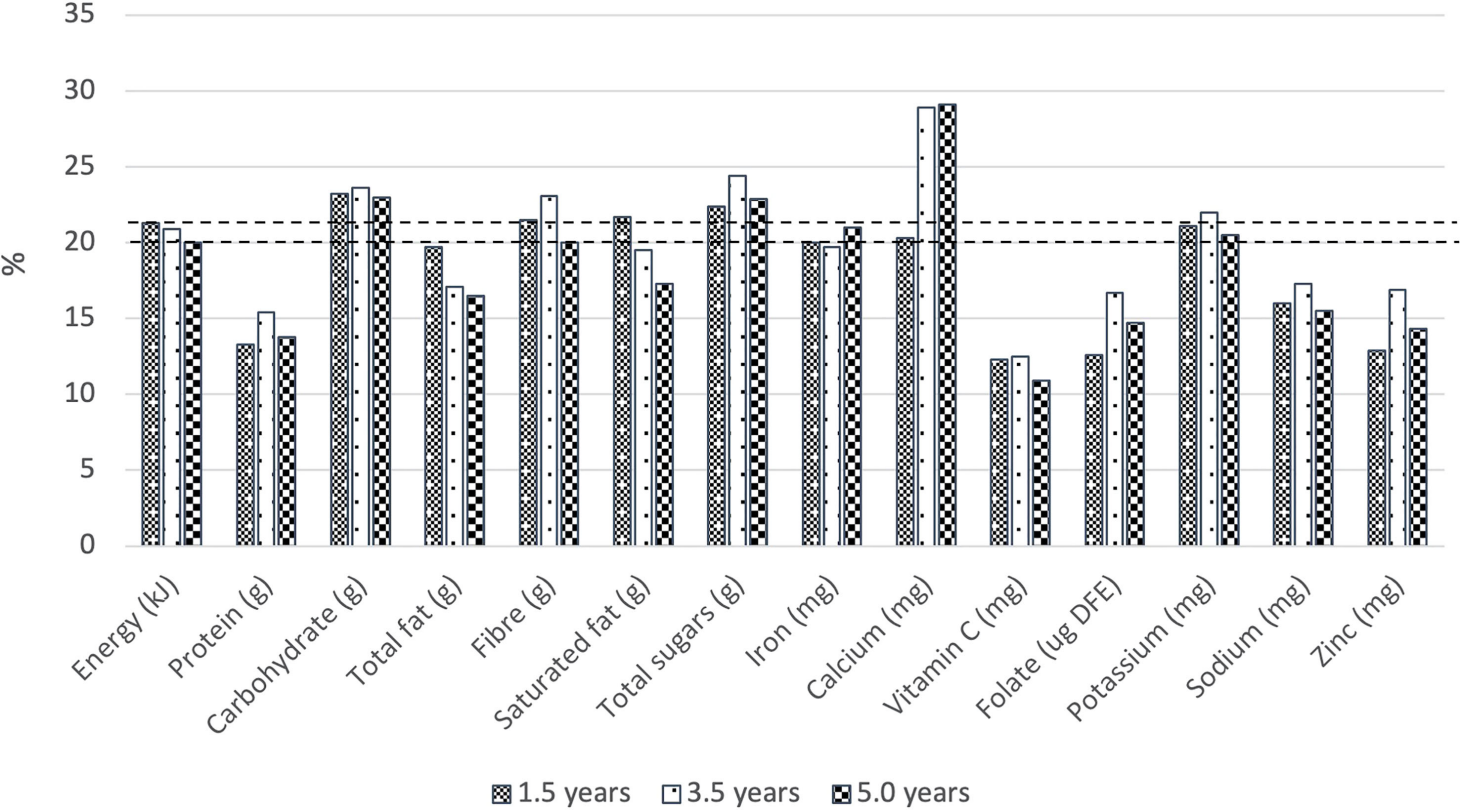
Contribution (%) of energy and nutrients at breakfast to daily energy and nutrient intakes by children aged 1.5 years (n=369), 3.5 years (n=244), and 5.0 years (n=240). Horizontal lines represent the contribution (20.0% - 21.3%) of energy intake from breakfast to total daily energy intake.

The contribution of energy intakes at breakfast ranged from 20.0% to 21.3% of daily energy intakes across the three ages (**Figure 1** and **Supplementary Table 5**). Carbohydrate, total sugars, calcium and potassium intakes at breakfast contributed proportionately more than 20% to total daily intakes whereas breakfast intakes of total fat, vitamin C, folate, sodium and zinc contributed less than 20% to total daily intakes. The contribution of calcium to daily intakes was highest at 5.0 years (29.1%) and 3.0 years (28.9%), compared with 1.5 years (20.3%).

##### 3.2.2.2 Contribution (%) to daily recommendations

In terms of macronutrient intakes, more than half (50.8%) of the EAR for protein and a fifth (20%) of the AI for fibre were consumed at breakfast across all three ages. The contribution of total energy from saturated fat across all three ages (11.9%-15.2%) at breakfast was higher compared to the Australian Dietary Guidelines for children and adolescents (no more than 10% of total energy from saturated fat) (45).

For all micronutrients of interest (i.e., iron, calcium, vitamin C, folate, sodium and zinc), children consumed more than 20% of the EAR or AI, with the exception of potassium. Sodium intakes were the highest, with children consuming approximately 40% of the maximum threshold of AI across all three ages (**Table 3**).

**Table 3:** Usual energy and nutrient intakes at breakfast and contribution (%) to recommended daily nutrient intakes.

Intakes of nutrients at breakfast	1.5 years (n=369)	^a^ Reference values of EAR (1-3 y)	Contribution of breakfast to EAR (%)	3.5 years (n=244)	^a^ Reference values of EAR (4-8 y)	Contribution of breakfast to EAR (%)	5.0 years (n=240)	^a^ Reference values of EAR (4-8 y)	Contribution of breakfast to EAR (%)
Energy (kJ)	951.07 (332.0)	EER:3800-4000 kJ/day	24.4	1108.0 (358.0)	EER:5800-6300 kJ/day	18.3	1180.0 (347.0)	EER:6500-7000 kJ/day	17.5
Macro-nutrients	Mean (SD)		%	Mean (SD)		%	Mean (SD)		%
Protein (g)	6.1 (3.4)	12g/day	50.8	8.3 (3.8)	16g/day	51.9	8.3 (3.5)	16g/day	51.9
% of energy from protein ^b^	16.0			16.0			16.0		
Carbohydrate (g)	30.0 (12.0)	None set	–	37.0 (12.0)	None set	–	40.0 (12.0)	None set	–
% of energy from carbohydrate ^c^	52.0			57.0			58.0		
Total fat (g)	7.5 (3.0)	None set	–	7.7 (3.6)	None set	–	8.1(3.4)	None set	–
% of energy from total fat ^d^	29.0			25.0			24.0		
Fibre (g)	2.8 (1.7)	AI:14g/day	20.0	3.7 (1.7)	AI:18g/day	20.6	3.6 (1.7)	AI:18g/day	20.0
% of energy from fibre ^e^	2.2			2.4			2.4		
Saturated fat (g)	3.9 (2.0)	None set	–	3.9 (2.3)	None set	–	3.8 (1.9)	None set	–
% of energy from saturated fat ^d^	15.2			13.0			11.9		
Total sugars (g)	15.0 (7.5)	None set	–	19.0 (8.2)	None set	–	19.0 (7.4)	None set	–
% of energy from total sugars	26.8			29.2			27.4		
Micronutrients	Mean (SD)		%	Mean (SD)		%	Mean (SD)		%
Iron (mg)	1.3 (1.3)	4mg/day	32.5	1.4 (1.4)	4mg/day	35.0	1.7 (1.5)	4mg/day	42.5
Calcium (mg)	152.0 (89.0)	360mg/day	42.2	210.0 (89.0)	520mg/day	40.4	222.0 (92.0)	520mg/day	42.7
Vitamin C (mg)	6.4 (8.9)	25mg/day	25.6	8.6 (13.0)	25mg/day	34.4	8.5 (12.0)	25mg/day	34.0
Folate (ug DFE)	32.0 (46.0)	120ug/day	26.7	51.0 (44.0)	160ug/day	31.9	50.0 (65.0)	160ug/day	31.3
Potassium (mg)	380.0 (155.0)	AI:2000mg/day	19.0	435.0 (156.0)	AI:2300mg/day	18.9	439.0 (165.0)	AI:2300mg/day	19.9
Sodium (mg)	174.0 (85.0)	AI: 200-400mg/day	43.5 ^f^	260.0 (94.0)	AI:300-600mg/day	43.3 ^f^	265.0 (130.0)	AI:300-600mg/day	44.2 ^f^
Zinc (mg)	0.8 (0.7)	2.5mg/day	32.0	1.2 (0.8)	3mg/day	40.0	1.1 (0.7)	3mg/day	36.7

EAR, Estimated Average Requirement; Al, Adequate Intake; EER, Estimated Energy Requirement.

a: According to Daily Nutrient Reference Values (NRV) for Australia and New Zealand, 2019; b: 17kJ for 1g of protein; c: 17kJ for 1g of carbohydrate.

d: 37kJ for 1g of fat and saturated fat; e: 8kJ for 1g of fibre; f: maximum threshold of AI.

### 3.3 Tracking of food group and nutrient intakes over time

There was low and moderate tracking of intakes for grains (0.16 ≤ r ≤ 0.48), discretionary foods (0.28 ≤ r ≤ 0.30), milk/alternatives (0.33 ≤ r ≤ 0.44) across the three ages (**Table 4**). There was inconsistent tracking of intakes for fruit, meat/alternatives, and wholegrains across the three ages. For most nutrients, tracking was found to be low or moderate between the different ages. The exception was for iron, which had low tracking between ages 1.5 and 3.5 years and 1.5 and 5 years, but high tracking (r=0.60, p<0.001) between ages 3.5 and 5.0 years.

**Table 4:** Tracking of the breakfast intakes of food groups and selected nutrients over time of children (aged 1.5-5.0 years) in the Melbourne Infant Feeding Activity and Nutrition Trial (InFANT) Program.

	1.5 years *vs* 3.5 years	1.5 years *vs* 5.0 years	3.5 years *vs* 5.0 years
	r^a^	r	r
Food groups			
Grains (g)	0.16*	0.23**	0.48***
Wholegrains (g)	0.30***	0.22***	0.06
Milk/alternatives (g)	0.44***	0.33***	0.41***
Fruit (g)	0.41***	0.07	0.50***
Meat/alternatives (g)	-0.04	-0.49***	-0.21**
Discretionary foods (kJ)	0.30***	0.30***	0.28***
Nutrients			
Energy (kJ)	0.25**	0.18*	0.31***
Protein (g)	0.23**	0.16*	0.29***
Carbohydrate (g)	0.26**	0.18*	0.32***
Total fat (g)	0.24**	0.18*	0.30***
Fibre (g)	0.26**	0.17*	0.31***
Saturated fat (g)	0.23**	0.15	0.28***
Total sugars (g)	0.22**	0.17*	0.31***
Iron (mg)	0.26***	0.21**	0.60***
Calcium (mg)	0.23**	0.15	0.28***
Vitamin C (mg)	0.21**	0.42***	0.17*
Folate (ug DFE)	0.24**	0.15	0.29***
Potassium (mg)	0.24**	0.16*	0.29***
Sodium (mg)	0.24**	0.14	0.29***
Zinc (mg)	0.25**	0.15	0.28***

a Pearson correlation of linear regression predicted residuals of food groups and nutrients at 1.5, 3.5, 5.0 years using age, gender, energy intake as covariates; <0.3 for slight tracking, 0.3-0.6 for moderate tracking, >0.6 for high tracking, consistent with previous approach (43). *p<0.05; **p<0.01; ***p<0.001.

## 4 Discussion

The present study provides important insights into breakfast consumption trends among young children (≤5 years of age) in Australia. This study is the first investigation of breakfast consumption including intakes of food groups and nutrients and their tracking over time across a range of ages between 1.5 and 5 years. This study found that this sample of young children in Australia; i) generally consumed grains, milk/alternatives and discretionary items at breakfast, with vegetables rarely consumed at breakfast at any age; ii) consumed at least 20% of the EAR or AI for the nutrients of interest at breakfast, with over 40% from sodium; iii) had intakes of saturated fat at breakfast that contributed marginallly more than the recommended 10% of total daily energy; and iv) had low or moderate tracking of most food groups and nutrients across the ages of 1.5, 3.5 and 5.0 years.

In our study, grain foods were commonly consumed at breakfast (namely breakfast cereals, bread and bread rolls) followed by milk/alternatives (namely cow’s milk or soy/oat alternative and yoghurt) across all three ages. These findings align with a previous German study (22), reporting that grain foods were common breakfast foods consumed by children aged 2-18 years. Among American children aged 2 to 5 years (25), milk/alternatives were the most consumed food at breakfast, followed by grains such as ready-to-eat cereals. In the UK, consumption of drinks or milk/alternatives such as tea, coffee, water, and semi-skimmed milk ranked highest for children aged 5–17 years, followed by high-fibre breakfast cereals (6). While the contribution of each of these food groups is important, the contribution of their joint consumption appears equally as important. Winiarska-Mieczan et al. (2016) identified school-aged children in Poland had a more favourable micronutrient intake when grain foods (i.e., Ready To Eat (RTE) cereals) and milk/alternatives (i.e. milk) were consumed together (46).

While different grains and milk/alternatives appear to be provided at breakfast, it would be desirable to have a more diverse combination of food groups at breakfast to enhance nutrient adequacy. Consuming at least three food groups at breakfast is suggested as a definition of a quality breakfast (47) as this can help close nutrient gaps and improve overall eating patterns.

The ADGs recommend that most of the grains consumed should be in the form of wholegrains (48), as fibre from wholegrains has been shown to improve appetite control, increase satiety, and enhance gut microbiota (49). Galea et al. (2017) found that approximately 70% of Australian children aged 2-18 years consumed wholegrains in a day (50). Similarly, in the UK (51), 73% of children aged 4-18 years consumed wholegrains during 7 days of dietary assessment. In our study, about three out of five 1.5-year-old children consumed wholegrains at breakfast, and wholegrains made up more than half of breakfast grain intakes at this age. However, the proportion of children consuming wholegrains decreased over time, with nearly half as many children consuming wholgrains at 5 years as at 1.5 years. While it is possible that children consumed wholegrains at later meals and snacks, breakfast provides an opportunity to consume wholegrain varieties of breakfast cereals and breads which are already commonly consumed at breakfast by children of all ages. The decline in wholegrain intake at breakfast observed across early childhood in the present study could be prevented among future generations by supporting parents to introduce age-appropriate wholegrain breakfast foods early in life to help foster liking and preference for these foods and to help maintain their intake as children age.

While few children in our study consumed meat/alternatives at breakfast, their protein intake was more than half the EAR. Given the foods most commonly consumed, this was likely coming from milk/alternatives. The main milk/alternatives consumed by children across all three ages was cow’s milk. Although the ADGs advise that children over age 2 years can consume reduced-fat milk (33), the majority of children consumed full-fat milk, with a small number of children consuming reduced-fat milk at ages 3.5 (6.1–9.0%) and 5 (5.0-6.7%) years. It may be beneficial to increase variety of breakfast foods by substituting milk (and cereal) with cheese or baked beans (and bread), while being conscious of the fat and sodium content of these foods (47). Breakfasts rich in protein can improve satiety and diet quality (52). However, the ideal amount and source of protein for breakfast remain unclear (53, 54).

Vegetables were rarely consumed during breakfast (≤1.4g mean intakes and <3% of children) across the three ages in our study. In Australia, only 6% of children aged 2-17 years of age meet the ADG recommended intakes of vegetables (55). Previous studies among older age groups have shown breakfast consumption increased the likelihood of meeting vegetable intake recommendations (56–58). In Spain, Lazzeri et al. (2013) (59) reported that vegetable intakes were more likely to be met by school-aged children who regularly consumed breakfast. Similarly, breakfast consumers among Australian and US adults were more likely to meet recommended guidelines for daily serves of vegetables (60, 61). Given that breakfast provides an opportunity to improve daily recommended vegetable intakes, vegetable consumption should be encouraged at this meal. However, the feasibility of incorporating vegetables into breakfast may be influenced by challenges such as preparation inconvenience, time constraints, or lack of acceptable options in the busy morning hours (56). To this end, simple, convenient and culturally acceptable approaches to facilitate the consumption of vegetables by young children should be considered and promoted. For instance, parents could be encouraged to provide wholemeal toast with sliced/mashed avocado (the most commonly consumed vegetable at breakfast in the present study) or cheese and sliced tomatoes (providing foods from three food groups). Additionally, parents could be encouraged to chop vegetables the night before making a quick breakfast vegetable omelette to overcome time pressures and inconvenience (and serving this on top of wholemeal toast could help maintain wholegrain intakes as well). Another option could be to make a vegetable-rich frittata that can be reheated and served for breakfast. Given that children are not used to vegetables being included in the breakfast meal, the intoduction of vegetables as a breakfast food may need to be done early in life so that children learn to accept these.

Breakfast can be considered a nutrient-rich eating occasion considering its contribution to total daily intakes. The International Breakfast Research Initiative (IBRI) defines a nutrent-rich breakfast as providing at least 20% of total daily intakes (except for sodium) (5, 20). Our study found that while breakfast contributed about 20% of total daily energy, this provided 20%-29.1% of total daily intake across all ages for carbohydrates, total sugars, calcium and potassium. Similarly, in the UK (6), the contribution of breakfast to total daily intakes for vitamin B, vitamin D, calcium, iodine and iron ranged from 20% to 41% across all age groups between 5 to 96 years, which is consistent with current available UK-based breakfast recommendations (over 20% of daily micronutrient intakes) (57). Notably, our study found a relatively high calcium contribution (20.3%-29.1% of daily calcium intake), believed to be driven by high intakes of milk/alternatives at breakfast. In fact, most of the calcium consumed among Australian children aged 2-11 years was from milk and alternatives (58). Furthermore, breakfast cereals, bread, flours, margarine, salt, snack bars, milk and plant-based milk alternatives, juices, and baby foods, which are frequently consumed by Australian children, are commonly fortified with calcium in Australia (31, 62). Consuming adequate calcium from an early age is associated with the prevention of osteoporosis (63), therefore the practice of providing calcium-rich foods and beverages at breakfast should be continued.

The present study has shown that children consumed at least 20% of the EAR or AI for all examined nutrients except for potassium at breakfast. This result is in line with previous studies which have suggested that at least 20% of recommended daily nutrient intakes should come from breakfast (47, 64). However, the relatively high contribution of sodium at breakfast to recommended daily intake for children across all ages (1.5 years: 43.5% of maximum AI, 3.5 years: 43.3% of maximum AI and 5.0 years: 44.2% of maximum AI) is concerning. This finding aligns with studies among children aged 6-17 years in Canada (5) and the US (38). It is well known that a high intake of sodium has a long-term effect on children’s taste, food preferences, and lifelong eating habits (63). High sodium intake observed in our study could be attributable to intakes of discretionary foods but could also be due to consumption of core foods such as breakfast cereals, meat and milk products (65–68). Discretionary foods consumed at breakfast were mostly toppings or spreads that were likely consumed on bread or toast. Australian food culture includes consumption of bread/toast with a yeast extract spread (“Vegemite”) (67) that is high in sodium (3300 mg/100 g) and is mainly consumed at breakfast in Australia (68). Vegemite was consumed at breakfast by 7.4% to 16.3% of young children in the present study. Given its cultural significance, it may be most feasible and appropriate to recommend that children consume salt-reduced Vegemite and/or less Vegemite at breakfast to reduce their sodium intake. Alternative topping and spread options (e.g. nut paste/spread, avocado, cheese with sliced tomatoes) at breakfast would also help lower sodium intakes and displace intakes of discretionary foods. Also, considering cereal and cereal products’ overall contributions to sodium are high, recommendations like replacing ultra-processed breakfast cereals with less processed options (e.g., oats) could be considered.

Australian Dietary Guidelines recommend that <10% of total energy should come from saturated fat (69), but our study showed a higher breakfast contribution of saturated fat (11.9% - 15.2% contribution of total energy at breakfast). Considering intakes of saturated fat at breakfast should be reduced, nut paste/spread or baked beans (on bread/toast) could be suggested as a breakfast recommendation to provide healthy unsaturated fats and lower amounts of saturated fat for breakfast. This could also be linked to a call for reduced-fat milk to occur at age 2.

There was low to moderate tracking of most food groups and nutrients, which may be explained by the stable transition in diet throughout early childhood. High tracking of iron intakes was evident between 3.5 years and 5.0 years. Similarly, micronutrient intake at breakfast has been shown to track over time in one UK study, with high-level tracking of fibre, folic acid, vitamin C and iodine intake at breakfast in children aged 4–10 years (70). These findings emphasize the importance of developing healthy eating habits from early childhood, as eating habits established in early life may track into adulthood (71). Nonetheless, in this study, inconsistent tracking of some food groups and nutrients such as fruit, wholegrains, meat/alternatives, folate, and saturated fat consumed at breakfast was also observed in early childhood. Moreover, the intakes of meat/alternatives decreased over time and tracked inconsistently between 1.5 and 3.5 years. This result may be related to the fact that busy morning hours can make it difficult for parents to cook meat/alternatives. The inconsistent tracking for some food and nutrient intakes highlights the complexity of breakfast intake in the early childhood period. Future qualitative research investigating how and why breakfast foods change across early childhood could enhance understanding of inconsistent tracking of food group and nutrient intakes at breakfast.

This study has a number of strengths. It is the first study to investigate food group and nutrient intakes at breakfast of children under the age of 5 years in Australia, and the tracking of intakes across three ages. Also, high quality dietary data collected *via* three 24hr recalls over three non-consecutive days enabled detailed evaluation of key food groups and nutrient intakes consumed at breakfast. Three 24hr recalls permitted the use of Multiple Source Method (MSM) (41) to obtain usual intakes of food groups and nutrients. Furthermore, repeated dietary intake data over three time points in early childhood allowed examination of breakfast consumption trends as well as longitudinal tracking over time in early childhood.

The present study also has some limitations. There is no standard definition of breakfast and the breakfast definition used in the current study differs from the definitions used other studies, which may limit direct comparisons. In addition, coding classification according to food groups, particularly for wholegrains, was a difficult task in diet evaluation and reporting because there is no standard definition of wholegrains. The present study made assumptions around the NRVs and ADGs because these recommendations do not specify breakfast intakes. For the micronutrients with only an AI available, the suitability of nutrient intakes should be carefully considered because the evidence base for AI is weaker than that of EAR and RDI (72). Furthermore, the InFANT study had a high proportion of tertiary-educated mothers, which may limit the generalisability of the findings to the wider Australian population. Given higher parental education may promote better dietary intakes for children (73), it is possible that the current study reported the best-case scenario for children’s breakfast intake. As such, it is important that we understand breakfast consumption habits of young children across different levels of education to help inform appropriate interventions.

## 5 Conclusion

This study provides evidence that breakfast is a nutrient-rich meal occasion for young Australian children aged 1.5-5 years. However, breakfast can be a contributor of higher intakes of sodium and saturated fat, which require early intervention. Some food group and nutrient intakes at breakfast showed stable tracking across early childhood, suggesting the importance of the early establishment of healthy eating habits. These findings could inform the development of specific nutrient-based breakfast guidelines to guide the provision of a healthy breakfast to young Australian children across the home and education setting. Future policy and practice strategies should focus on increasing vegetable intake while reducing saturated fat and sodium intake at breakfast.

## Data availability statement

The original contributions presented in the study are included in the article/**Supplementary Material**. Further inquiries can be directed to the corresponding author.

## Ethics statement

The InFANT study has ethical approval from the Deakin University Ethics committee (ID number: EC 175-2007) and the Australian Victorian Office for Children (Ref: CDF/07/1138). Written informed consent to participate in this study was provided by the participants’ legal guardian/next of kin.

## Author contributions

Conceptualization: KL, MZ, SP. Data curation: KL, MZ, SP. Formal analysis: KL, SP. Project administration: KL. Resources: KC, KL. Software: KL, SP. Supervision: KL, PL, MZ, KC. Writing original draft: SP. Writing review and editing: KL, PL, MZ, KC. All authors contributed to the article and approved the submitted version.

## References

[1] Centre for Community Child Health. The first thousand days. (2017). Available at: https://www.rch.org.au/uploadedFiles/Main/Content/ccchdev/CCCH-The-First-Thousand-Days-An-Evidence-Paper-September-2017.pdf.

[2] Koletzko B Brands B Poston L Godfrey K Demmelmair H. Early nutrition programming of long-term health. Proc Nutr Soc (2012) 71:371–8. doi: 10.1017/S0029665112000596 22703585

[3] Schwartz C Scholtens PA Lalanne A Weenen H Nicklaus S. Development of healthy eating habits early in life. review of recent evidence and selected guidelines. Appetite (2011) 57:796–807. doi: 10.1016/j.appet.2011.05.316 21651929

[4] Hanson M Fall C Robinson S Baird J. Early life nutrition and lifelong health. (2009). Available at: https://www.researchgate.net/profile/Caroline-Seddon/publication/281282761_Early_life_nutrition_and_lifelong_health_BMA_February_2009/links/55df1fe908ae79830bb6fb9a/Early-life-nutrition-and-lifelong-health-BMA-February-2009.pdf.

[5] Barr SI Vatanparast H Smith J. Breakfast in Canada: prevalence of consumption, contribution to nutrient and food group intakes, and variability across tertiles of daily diet quality. a study from the international breakfast research initiative. Nutrients (2018) 10:985. doi: 10.3390/nu10080985 30060534 PMC6116091

[6] Gaal S Kerr MA Ward M McNulty H Livingstone MBE. Breakfast consumption in the UK: patterns, nutrient intake and diet quality. a study from the international breakfast research initiative group. Nutrients (2018) 10:999. doi: 10.3390/nu10080999 30061542 PMC6115898

[7] Dubois L Girard M Potvin Kent M Farmer A Tatone-Tokuda F. Breakfast skipping is associated with differences in meal patterns, macronutrient intakes and overweight among pre-school children. Public Health Nutr (2009) 12:19–28. doi: 10.1017/S1368980008001894 18346309

[8] Williams P. Breakfast and the diets of Australian children and adolescents: an analysis of data from the 1995 national nutrition survey. Int J Food Sci Nutr (2007) 58:201–16. doi: 10.1080/09637480701198075 17514538

[9] Fayet-Moore F McConnell A Tuck K Petocz P. Breakfast and breakfast cereal choice and its impact on nutrient and sugar intakes and anthropometric measures among a nationally representative sample of Australian children and adolescents. Nutrients 9 (2017) 9:1045. doi: 10.3390/nu9101045 PMC5691662 28934111

[10] Grieger JA Kim S Cobiac L. Where do Australian children get their dietary fibre? a focus on breakfast food choices. Nutr Dietetics (2013) 70:132–8. doi: 10.1111/j.1747-0080.2012.01640.x

[11] Smith KJ Breslin MC McNaughton SA Gall SL Blizzard L Venn AJ. Skipping breakfast among Australian children and adolescents; findings from the 2011–12 national nutrition and physical activity survey. John Wiley & Sons, Australian and New Zealand journal of public health (2017) 41:572–578. 10.1111/1753-6405.12715 28898562

[12] Grieger J Cobiac L. Comparison of dietary intakes according to breakfast choice in Australian boys. Eur J Clin Nutr (2012) 66:667–72. doi: 10.1038/ejcn.2011.220 22234045

[13] Fayet-Moore F Kim J Sritharan N Petocz P. Impact of breakfast skipping and breakfast choice on the nutrient intake and body mass index of Australian children. (2016) 8:487. doi: 10.3390/nu8080487 PMC4997400 27517957

[14] Kosti RI Panagiotakos DB Zampelas A Mihas C Alevizos A Leonard C. The association between consumption of breakfast cereals and BMI in schoolchildren aged 12-17 years: the VYRONAS study. Public Health Nutr (2008) 11:1015–21. doi: 10.1017/S1368980007001437 18093352

[15] Sincovich A Moller H Smithers L Brushe M Lassi ZS Brinkman SA. Prevalence of breakfast skipping among children and adolescents: a cross-sectional population level study. BMC Pediatr (2022) 22:1–10. doi: 10.1186/s12887-022-03284-4 35459164 PMC9034546

[16] Koca T Akcam M Serdaroglu F Dereci S. Breakfast habits, dairy product consumption, physical activity, and their associations with body mass index in children aged 6-18. Eur J Pediatr (2017) 176:1251–7. doi: 10.1007/s00431-017-2976-y 28799014

[17] Fagt S Matthiessen J Thyregod C Kørup K Biltoft-Jensen A. Breakfast in denmark. prevalence of consumption, intake of foods, nutrients and dietary quality. a study from the international breakfast research initiative. Nutrients (2018) 10:1085. doi: 10.3390/nu10081085 30110931 PMC6116167

[18] Bellisle F Hébel P Salmon-Legagneur A Vieux F. Breakfast consumption in French children, adolescents, and adults: a nationally representative cross-sectional survey examined in the context of the international breakfast research initiative. Nutrients (2018) 10:1056. doi: 10.3390/nu10081056 30096946 PMC6115921

[19] Ruiz E Ávila JM Valero T Rodriguez P Varela-Moreiras G. Breakfast consumption in Spain: patterns, nutrient intake and quality. findings from the ANIBES study, a study from the international breakfast research initiative. Nutrients (2018) 10:1324. doi: 10.3390/nu10091324 30231551 PMC6165504

[20] Gibney MJ Barr SI Bellisle F Drewnowski A Fagt S Livingstone B. Breakfast in human nutrition: the international breakfast research initiative. Nutrients (2018) 10:559. doi: 10.3390/nu10050559 29723985 PMC5986439

[21] Murakami K Livingstone MBE Fujiwara A Sasaki S. Breakfast in Japan: findings from the 2012 national health and nutrition survey. Nutrients (2018) 10:1551. doi: 10.3390/nu10101551 30347762 PMC6212985

[22] Alexy U Wicher M Kersting M. Breakfast trends in children and adolescents: frequency and quality. Public Health Nutr (2010) 13:1795–802. doi: 10.1017/S1368980010000091 20236559

[23] Alsharairi NA Somerset SM. Skipping breakfast in early childhood and its associations with maternal and child BMI: a study of 2-5-year-old Australian children. Eur J Clin Nutr (2016) 70:450–455. doi: 10.1038/ejcn.2015.184 26508462

[24] Smith KJ Breslin MC McNaughton SA Gall SL Blizzard L Venn AJ. Skipping breakfast among Australian children and adolescents; findings from the 2011–12 national nutrition and physical activity survey. Aust New Z J Public Health (2017) 41:572–8. doi: 10.1111/1753-6405.12715 28898562

[25] Terry AL Wambogo E Ansai N Ahluwalia N. Breakfast Intake Among Children and Adolescents: United States, 2015-2018. NCHS Data Brief. (2020), 1–8. Available at: https://stacks.cdc.gov/view/cdc/95311/cdc_95311_DS1.pdf. 33054919

[26] Campbell K Hesketh K Crawford D Salmon J Ball K McCallum Z. The infant feeding activity and nutrition trial (INFANT) an early intervention to prevent childhood obesity: cluster-randomised controlled trial. BMC Public Health (2008) 8:1–9. doi: 10.1186/1471-2458-8-103 18373877 PMC2346474

[27] Campbell KJ Lioret S McNaughton SA Crawford DA Salmon J Ball K. A parent-focused intervention to reduce infant obesity risk behaviors: a randomized trial. Pediatrics (2013) 131:652–60. doi: 10.1542/peds.2012-2576 23460688

[28] Hesketh KD Salmon J McNaughton SA Crawford D Abbott G Cameron AJ. Long-term outcomes (2 and 3.5 years post-intervention) of the INFANT early childhood intervention to improve health behaviors and reduce obesity: cluster randomised controlled trial follow-up. Int J Behav Nutr Phys Activity (2020) 17:95. doi: 10.1186/s12966-020-00994-9 PMC7382091 32711523

[29] Lacy KE Spence AC McNaughton SA Crawford DA Wyse RJ Wolfenden L. Home environment predictors of vegetable and fruit intakes among Australian children aged 18 months. Appetite (2019) 139:95–104. doi: 10.1016/j.appet.2019.04.009 30991083

[30] Conway JM Ingwersen LA Vinyard BT Moshfegh AJ. Effectiveness of the US department of agriculture 5-step multiple-pass method in assessing food intake in obese and nonobese women. Am J Clin Nutr (2003) 77:1171–8. doi: 10.1093/ajcn/77.5.1171 12716668

[31] Food Standards Australia New Zealand. AUSNUT 2007. Australia New Zealand: Food Standards (2007).

[32] Atkins LA McNaughton SA Campbell KJ Szymlek-Gay EA. Iron intakes of Australian infants and toddlers: findings from the Melbourne infant feeding, activity and nutrition trial (InFANT) program. Br J Nutr (2016) 115:285–93. doi: 10.1017/S0007114515004286 26571345

[33] National Health and Medical Research Council (NHMRC). Australian Dietary guidelines. Canberra: Commonwealth of Australia (2013).

[34] Mathews R Chu Y. Global review of whole grain definitions and health claims. Nutr Rev (2020) 78:98–106. doi: 10.1093/nutrit/nuz055 32728741

[35] Grains&Legumes Nutrition Council (GLNC). Whole grain ingredient content claims. (2021). Available at: https://www.glnc.org.au/codeofpractice/whole-grain-ingredient-content-claims/.

[36] Australian Bureau of Statistics (ABS). Australian Health survey: users’ guide, 2011-13. Discretionary Food (2014). Available at: https://www.health.gov.au/topics/food-and-nutrition?utm_source=health.gov.au&utm_medium=callout-auto-custom&utm_campaign=digital_transformation.

[37] Australian Government: National Health and Medical Research Council. Nutrient reference values. Nutrient Reference Values for Australia and New Zealand (2020). Available at: https://www.nhmrc.gov.au/sites/default/files/images/nutrient-refererence-dietary-intakes.pdf.

[38] Vieux F Maillot M Rehm CD Drewnowski A. Designing optimal breakfast for the united states using linear programming and the NHANES 2011–2014 database: a study from the international breakfast research initiative (IBRI). Nutrients (2019) 11:1374. doi: 10.3390/nu11061374 31248096 PMC6627424

[39] Leech RM Worsley A Timperio A McNaughton SA. Characterizing eating patterns: a comparison of eating occasion definitions. Am J Clin Nutr (2015) 102:1229–37. doi: 10.3945/ajcn.115.114660 26447152

[40] Leech RM Spence AC Lacy KE Zheng M Timperio A McNaughton SA. Characterizing children's eating patterns: does the choice of eating occasion definition matter? Int J Behav Nutr Phys Act (2021) 18:165. doi: 10.1186/s12966-021-01231-7 34923993 PMC8684678

[41] Harttig U Haubrock J Knüppel S Boeing H. The MSM program: web-based statistics package for estimating usual dietary intake using the multiple source method. Eur J Clin Nutr (2011) 65:S87–91. doi: 10.1038/ejcn.2011.92 21731011

[42] Lioret S McNaughton S Spence A Crawford D Campbell K. Tracking of dietary intakes in early childhood: the Melbourne InFANT program. Eur J Clin Nutr (2013) 67:275–81. doi: 10.1038/ejcn.2012.218 PMC5385208 23321573

[43] Campbell KJ Abbott G Zheng M McNaughton SA. Early life protein intake: food sources, correlates, and tracking across the first 5 years of life. J Acad Nutr Dietetics (2017) 117:1188–1197. e1. doi: 10.1016/j.jand.2017.03.016 28527745

[44] Twisk J Kemper H Van Mechelen W Post G. Tracking of risk factors for coronary heart disease over a 14-year period: a comparison between lifestyle and biologic risk factors with data from the Amsterdam growth and health study. Am J Epidemiol (1997) 145:888–98. doi: 10.1093/oxfordjournals.aje.a009048 9149660

[45] National Health and Medical Research Council (NHMRC). Dietary guidelines for children and adolescents in Australia: incorporationg the infant feeding guidelines for health workers. Canbera: Commonwealth of Australia (2003).

[46] Winiarska-Mieczan A Kwiecień M Kwiatkowska K Krusiński R. Breakfast cereal as a source of sodium, potassium, calcium and magnesium for school-age children. J Elemntology (2016) 21:571–84. doi: 10.5601/jelem.2015.20.1.763

[47] O'Neil C Byrd-Bredbenner C Hayes D Jana L Klinger S Stephenson-Martin S. The role of breakfast in health: definition and criteria for a quality breakfast. J Acad Nutr Dietetics (2014) 114:S8–S26. doi: 10.1016/j.jand.2014.08.022 25458994

[48] National Health and Medical Research Council (NHMRC). Australian Dietary guidelines. Guide to Healthy Eating. (2013). Available at https://www.nhmrc.gov.au/adg#block-views-block-file-attachments-content-block-1.

[49] Cooper DN Martin RJ Keim NL. Does whole grain consumption alter gut microbiota and satiety? In Healthcare MDPI (2015) 3:364–92. doi: 10.3390/healthcare3020364 PMC4939539 27417768

[50] Galea LM Beck EJ Probst YC Cashman CJ. Whole grain intake of australians estimated from a cross-sectional analysis of dietary intake data from the 2011–13 Australian health survey. Public Health Nutr (2017) 20:2166–72. doi: 10.1017/S1368980017001082 PMC10261361 28592344

[51] Thane C Jones A Stephen A Seal C Jebb S. Whole-grain intake of British young people aged 4–18 years. Br J Nutr (2005) 94:825–31. doi: 10.1079/BJN20051557 16277788

[52] Leidy HJ Ortinau LC Douglas SM Hoertel HA. Beneficial effects of a higher-protein breakfast on the appetitive, hormonal, and neural signals controlling energy intake regulation in overweight/obese,”breakfast-skipping,” late-adolescent girls. Am J Clin Nutr (2013) 97:677–88. doi: 10.3945/ajcn.112.053116 PMC3718776 23446906

[53] Nicklas TA Reger C Myers L O’Neil C. Breakfast consumption with and without vitamin-mineral supplement use favorably impacts daily nutrient intake of ninth-grade students. J Adolesc Health (2000) 27:314–21. doi: 10.1016/S1054-139X(00)00113-0 11044703

[54] Apolzan JW Carnell NS Mattes RD Campbell WW. Inadequate dietary protein increases hunger and desire to eat in younger and older men. J Nutr (2007) 137:1478–82. doi: 10.1093/jn/137.6.1478 PMC2259459 17513410

[55] Australian Institute of Health and Welfare(AIHW). Australia's children. (2020). doi: 10.25816/5ebca4d0fa7dd

[56] Videon TM Manning CK. Influences on adolescent eating patterns: the importance of family meals. J Adolesc Health (2003) 32:365–73. doi: 10.1016/S1054-139X(02)00711-5 12729986

[57] Crawley H. Eating well at school - nutritional and practical guidelines the Caroline walker trust. (2005). Available at: https://www.cwt.org.uk/wp-content/uploads/2014/07/EatingWellatSchool.pdf.

[58] Williams P. Breakfast and the diets of Australian children and adolescents: an analysis of data from the 1995 National Nutrition Survey. International Journal of Food Sciences and Nutrition. (2007) 1998:201–216. Available at: https://www.abs.gov.au/ausstats/abs%40.nsf/lookup/95e87fe64b144fa3ca2568a9001393c0. 10.1080/09637480701198075 17514538

[59] Lazzeri G Pammolli A Azzolini E Simi R Meoni V de Wet DR. Association between fruits and vegetables intake and frequency of breakfast and snacks consumption: a cross-sectional study. Nutr J (2013) 12:123. doi: 10.1186/1475-2891-12-123 23981379 PMC3765730

[60] Fayet-Moore F McConnell A Cassettari T Petocz P. Breakfast choice is associated with nutrient, food group and discretionary intakes in australian adults at both breakfast and the rest of the day. Nutrients (2019) 11:175. doi: 10.3390/nu11010175 30650604 PMC6356876

[61] Deshmukh-Taskar PR Radcliffe JD Liu Y Nicklas TA. Do breakfast skipping and breakfast type affect energy intake, nutrient intake, nutrient adequacy, and diet quality in young adults? NHANES 1999–2002. J Am Coll Nutr (2010) 29:407–18. doi: 10.1080/07315724.2010.10719858 21041816

[62] Food Standards Australia New Zealand (FSANZ). Nutrition and fortification. (2016). Available at: https://www.foodstandards.gov.au/consumer/nutrition/Pages/default.aspx.

[63] Greer FR Krebs NF. Nutrition, optimizing bone health and calcium intakes of infants, children, and adolescents. Pediatrics (2006) 117:578–85. doi: 10.1542/peds.2005-2822 16452385

[64] Taylor CL Stallings VA. Institute of Medicine. Nutrition Standards and Meal Requirements for National School Lunch and Breakfast Programs: Phase I. Proposed Approach for Recommending Revisions (2008). Washington, DC: The National Academies Press. doi: 10.17226/12512 25009924

[65] O'Halloran SA Grimes CA Lacy KE Nowson CA Campbell KJ. Dietary sources and sodium intake in a sample of Australian preschool children. BMJ Open (2016) 6:e008698. doi: 10.1136/bmjopen-2015-008698 PMC4746469 26846894

[66] Campbell KJ Hendrie G Nowson C Grimes CA Riley M Lioret S. Sources and correlates of sodium consumption in the first 2 years of life. J Acad Nutr Dietetics (2014) 114:1525–1532.e2. doi: 10.1016/j.jand.2014.04.028 25022834

[67] Johnson BJ Bell LK Zarnowiecki D Rangan AM Golley RK. Contribution of discretionary foods and drinks to Australian children’s intake of energy, saturated fat, added sugars and salt. Children (2017) 4:104. doi: 10.3390/children4120104 29194425 PMC5742749

[68] Grimes CA Riddell LJ Campbell KJ Beckford K Baxter JR He FJ. Dietary intake and sources of sodium and potassium among Australian schoolchildren: results from the cross-sectional salt and other nutrients in children (SONIC) study. BMJ Open (2017) 7:e016639. doi: 10.1136/bmjopen-2017-016639 PMC5665305 29084791

[69] World Health Organization Healthy diet. (2020).

[70] Coulthard JD Palla L Pot GK. Breakfast consumption and nutrient intakes in 4-18-year-olds: UK national diet and nutrition survey rolling programme (2008-2012). Br J Nutr (2017) 118:280–90. doi: 10.1017/S0007114517001714 28814349

[71] Ashcroft J Semmler C Carnell S van Jaarsveld CH Wardle J. Continuity and stability of eating behaviour traits in children. Eur J Clin Nutr (2008) 62:985–90. doi: 10.1038/sj.ejcn.1602855 17684526

[72] Australian Government Department of Health and Aged Care. Methodological framework for the review of nutrient reference values. (2015). Available at: https://www.eatforhealth.gov.au/sites/default/files/2022-10/Final_NRV_Methodological_Framework_v2.0_0.pdf.

[73] Gibbs HD Kennett AR Kerling EH Yu Q Gajewski B Ptomey LT. Assessing the nutrition literacy of parents and its relationship with child diet quality. J Nutr Educ Behav (2016) 48:505–509. e1. doi: 10.1016/j.jneb.2016.04.006 27216751 PMC4931947

