# Supplementary material for: Breakfast consumption trends among young Australian children aged up to 5 years: results from InFANT program
Source: Front Endocrinol (Lausanne). 2023 Aug 10;14:1154844. doi: 10.3389/fendo.2023.1154844 (PMC10448523; doi:10.3389/fendo.2023.1154844)
Supplement: Supplementary file 1 [file DataSheet_1.docx]

**Supplementary Table 1. Food group classification ^a^**

| No | Food groups | Items included |
| --- | --- | --- |
| 1 | Grains (cereal) foods | Cereals and cereal products |
| 2 | Wholegrains | Sub-items as part of grains Including whole wheat, wheat bran, wholemeal flour, mixed grain, oats, rye flour (dark), and rice (brown) |
| 3 | Milk products / alternatives | Milk products and dishes (e.g. milk, yogurt and cheese) |
| 4 | Meat/alternatives | Meat, poultry, and game products and dishes |
| 5 | Fruits | Fresh, frozen, and 100% fruit juices |
| 6 | Vegetables | Fresh, frozen, dried vegetable products and dishes |
| 7 | Discretionary foods | Including sweet cereals, sweet drinks, condensed milk, sugar, syrups, savoury sauces, vegemite, and processed meats |
| 8 | Infant cereal products | Infant cereals and infant rusk |
| 9 | Infant formulae | Infant formulae and human breast milk |

^a^ According to the 2013 Australian Dietary Guidelines (ADG 2013)


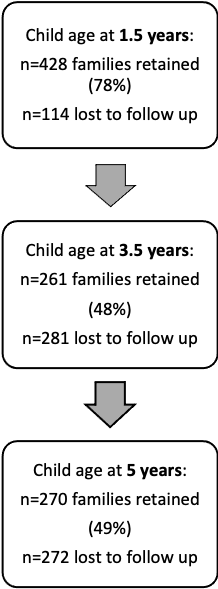

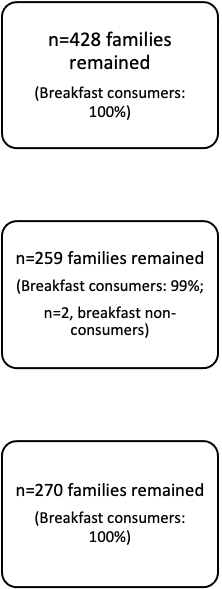

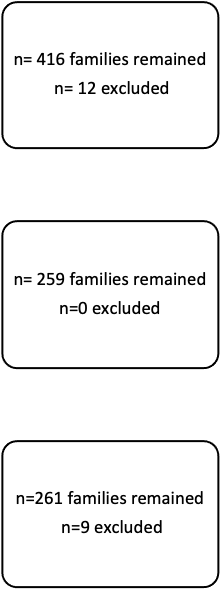

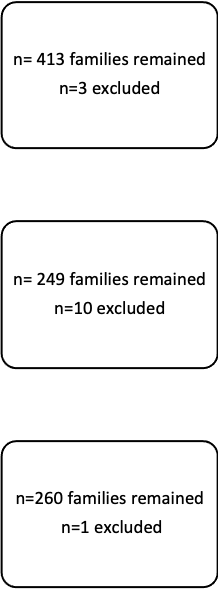

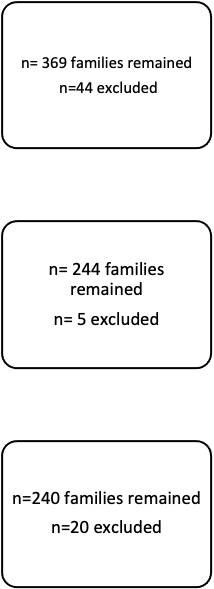


**Melbourne InFANT Program Sample**

**(n=542 at baseline)**

**Breakfast consumers**

**First-time parent**

**Energy intake ± 3SD**

**Three, 24-hour recalls**


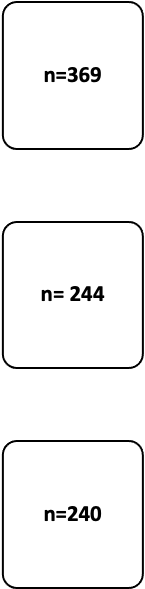


**Final sample**

**for analysis**

**Supplementary Figure 1. Flowchart outlining the sample from the Melbourne Infant Activity and Nutrition Trial (InFANT) study included at each time point and for each analysis among breakfast consumers**

**Supplementary Table 2. Usual intakes of energy and selected nutrients at breakfast of children (aged 1.5 (n=369), 3.5 (n=244) and 5.0 (n=240) years) in the control and intervention groups of the Melbourne Infant Feeding Activity and Nutrition Trial (InFANT) Program.**

|  | **1.5 years** |  |  | **3.5 years** |  |  | **5.0 years** |  |  |
| --- | --- | --- | --- | --- | --- | --- | --- | --- | --- |
|  | **Control** | **Intervention** | **P** | **Control** | **Intervention** | **P** | **Control** | **Intervention** | **P** |
| Energy (kJ) | 964.05 | 1012.11 | 0.25 | 1074.99 | 1127.43 | 0.96 | 1171.86 | 1194.77 | 0.56 |
| Protein (g) | 9.17 | 9.72 | 0.20 | 10.34 | 11.09 | 0.20 | 11.30 | 11.70 | 0.49 |
| Carbohydrate (g) | 30.37 | 32.33 | 0.19 | 38.13 | 37.71 | 0.82 | 39.31 | 42.01 | 0.17 |
| Total fat (g) | 7.53 | 7.75 | 0.69 | 7.84 | 7.70 | 0.81 | 8.65 | 8.01 | 0.30 |
| Fibre (g) | 2.84 | 3.05 | 0.35 | 3.46 | 3.51 | 0.84 | 3.48 | 3.89 | 0.12 |
| Saturated fat (g) | 3.91 | 4.19 | 0.25 | 3.78 | 3.86 | 0.81 | 3.86 | 3.96 | 0.73 |
| Total sugars (g) | 14.67 | 16.15 | 0.12 | 20.19 | 18.65 | 0.24 | 19.30 | 19.88 | 0.63 |
| Iron (mg) | 2.27 | 2.49 | 0.19 | 2.31 | 2.56 | 0.23 | 2.53 | 2.90 | 0.10 |
| Calcium (mg) | 192.50 | 202.31 | 0.39 | 203.53 | 221.42 | 0.20 | 216.33 | 231.57 | 0.30 |
| Vitamin C (mg) | 7.77 | 5.53 | 0.07 | 9.32 | 8.22 | 0.60 | 8.88 | 8.45 | 0.78 |
| Folate (ug DFE) | 86.63 | 80.83 | 0.39 | 98.01 | 90.43 | 0.31 | 106.89 | 109.19 | 0.84 |
| Potassium (mg) | 380.70 | 407.94 | 0.17 | 434.96 | 451.17 | 0.51 | 433.62 | 466.97 | 0.18 |
| Sodium (mg) | 180.22 | 182.28 | 0.86 | 231.74 | 228.23 | 0.84 | 278.68 | 258.29 | 0.39 |
| Zinc (mg) | 1.66 | 1.77 | 0.26 | 1.73 | 1.90 | 0.14 | 1.78 | 2.00 | 0.05 |

*p<0.05; **p<0.01; ***p<0.001

**Supplementary Table3. Usual intakes (g/three non-consecutive days) of food groups at breakfast of children (aged 1.5 (n=369), 3.5 (n=244) and 5.0 (n=240) years) in the control and intervention groups of the Melbourne Infant Feeding Activity and Nutrition Trial (InFANT) Program.**

|  | **1.5years** |  |  | **3.5years** |  |  | **5.0years** |  |  |
| --- | --- | --- | --- | --- | --- | --- | --- | --- | --- |
|  | **Control** | **Intervention** | **P** | **Control** | **Intervention** | **P** | **Control** | **Intervention** | **P** |
| Grains | 38.69 | 36.36 | 0.47 | 55.41 | 50.94 | 0.37 | 60.17 | 58.67 | 0.75 |
| Wholegrains | 24.70 | 25.38 | 0.98 | 20.16 | 23.46 | 0.96 | 11.98 | 13.23 | 0.87 |
| Milk/alternatives | 132.64 | 146.76 | 0.14 | 135.18 | 156.37 | 0.06 | 127.74 | 153.16 | 0.02* |
| Meat/alternatives | 2.48 | 2.27 | 0.78 | 3.44 | 3.39 | 0.96 | 4.16 | 4.81 | 0.58 |
| Fruit | 22.24 | 23.45 | 0.69 | 35.10 | 28.52 | 0.73 | 32.02 | 30.37 | 0.77 |
| Vegetable | 0.48 | 0.79 | 0.70 | 0.41 | 0.52 | 0.92 | 1.38 | 1.51 | 0.89 |
| Discretionary food (kJ) | 33.76 | 35.86 | 0.46 | 71.38 | 72.48 | 0.69 | 91.25 | 92.35 | 0.95 |
| Infant cereal | 0.79 | 0.82 | 0.98 | - | - | - | - | - | - |
| Infant formula | 15.87 | 16.12 | 0.87 | 2.62 | 2.68 | 0.85 | 1.58 | 1.61 | 0.78 |

*p<0.05; **p<0.01; ***p<0.001

**Supplementary Table 4. Percentage children consuming sub-food group items per major food groups at each age ^a)^**

| Food group ^b)^ | Rank ^c)^ | Food Items | | |
| --- | --- | --- | --- | --- |
|  |  | **1.5 years (n=369)** | **3.5 years (n=244)** | **5.0 years (n=240)** |
| Grains | 1 | Breakfast cereal (61.2% - 64.0%) ^d)^ | Breakfast Cereal (70.1% - 72.5%) | Breakfast Cereal (74.6% – 78.3%) |
|  | 2 | Bread and bread rolls (15.7% - 17.1%) | Bread and bread rolls (9.8% - 14.8%) | Bread and bread rolls (13.3% - 17.1%) |
| Whole grains | 1 | Mixed grain cereal (54.5% - 55.8%) | Mixed grain cereal (49.6% - 52.0%) | Mixed grain cereal (29.2% - 30.8%) |
|  | 2 | Wholemeal bread (1.9% - 3.8%) | Wholemeal bread (2.5% - 4.9%) | Wholemeal bread (4.2% - 5.0%) |
| Milk products/  alternatives | 1 | Milk, Cow, Full Fat (71.8%-73.2%) | Milk, Cow, Full Fat (62.7%-63.5%) | Milk, Cow, Full Fat (57.5%-65.0%) |
|  | 2 | Yogurt (6.5%-8.4%) | Milk, Cow, Reduced fat (6.1%-9.0%) | Milk, Cow, Reduced fat (5.0%-6.7%) |
|  | 3 | Soy / oat milk (2.4%-4.6%) | Yoghurt (4.5%-5.7%) | Soy / oat milk (3.8%-5.0%) |
| Meat/  alternatives | 1 | Eggs (2.7% - 3.5%) | Eggs (2.9% - 4.1%) | Eggs (3.3% - 5.4%) |
|  | 2 | Meat/ poultry (0.0% -2.7%) | - | Meat/ poultry (0.0% -4.2%) |
| Fruits | 1 | Fresh fruit (25.5% - 32.2%) | Fresh fruit (19.3% - 21.3%) | Fresh fruit (15.8% - 18.3%) |
|  | 2 | Dried, canned fruit (3.3% - 4.3%) | Fruit juice (7.0% - 9.4%) | Fruit juice (7.1% - 9.6%) |
|  | 3 | Fruit juice (1.9% - 3.5%) | Dried, canned fruit (4.9% - 6.1%) | Dried, canned fruit (2.9% - 3.8%) |
| Vegetables | 1 | Avocado (0.5% - 1.4%) | Avocado (0.8% - 2.9%) | Avocado (1.3% - 2.1%) |
|  | 2 | Tomato (0.8%) | - | Tomato, cherry, raw (0.8%) |
|  | 3 | Mushroom (0.5%) | - | - |
| Discretionary items | 1 | Vegemite (14.1% - 16.3%) | Honey, Sugar, Syrup (17.2% - 19.3%) | Honey, Sugar, Syrups (21.3% - 22.9%) |
|  | 2 | Honey, Sugar, Syrup (7.6% - 9.8%) | Vegemite (7.4% - 11.1%) | Vegemite (8.8% - 11.7%) |
|  | 3 | Sweet drinks (1.1% - 1.9%) | Sweet drinks (3.3% - 4.1%) | Sweet drinks (3.3% - 5.0%) |
| Infant cereal products | 1 | Infant cereals (1.9%-3.5%) | - | - |
|  | 2 | Infant rusk (0% -0.3%) | - | - |
| Infant formulae | 1 | Human Breast Milk (6.2%) | Toddler Formula, Milk Based (1.2%) | Toddler Formula, Milk Based (0.4%-0.8%)  -  - |
|  | 2 | Toddler Formula, Milk Based (3.0% - 3.8%) | Infant Formulae (0.4%) |  |
|  | 3 | Infant Formulae (2.4%-2.7%) | - |  |

a) Australian Food, Supplement and Nutrient Database (AUSNUT 2007); b) Major food group; c) Sub-major food group, sub-major food group defined as ‘consuming (yes, no)’ so some food group have 3 items while others only have 2 items; d) Brackets present the range for % of total children consuming each food item over three 24-hour dietary recalls.

**Supplementary Table 5. Contribution of energy and nutrients from breakfast as a proportion (%) of daily intakes by age groups ^a^**

|  | **1.5 years**  **(n=369)** | | | **3.5 years**  **(n=244)** | | | **5.0 years**  **(n=240)** | | |
| --- | --- | --- | --- | --- | --- | --- | --- | --- | --- |
|  | **Breakfast**  **Intake** | **Daily**  **Intake** | **Contribution of breakfast to daily intakes** | **Breakfast**  **Intake** | **Daily**  **Intake** | **Contribution of breakfast to daily intakes** | **Breakfast**  **Intake** | **Daily**  **Intake** | **Contribution of breakfast to daily intakes** |
|  | **Mean (SD)** | **Mean (SD)** | **%** | **Mean (SD)** | **Mean (SD)** | **%** | **Mean (SD)** | **Mean (SD)** | **%** |
| Energy (kJ) | 951.0 (332.0) | 4448.0 (652.0) | 21.3 | 1108.0 (358.0) | 5314.0 (869.0) | 20.9 | 1180.0 (347.0) | 5889.0 (1004.0) | 20.0 |
| Protein (g) | 6.1 (3.4) | 46.0 (8.5) | 13.3 | 8.3 (3.8) | 54.0 (10.0) | 15.4 | 8.3 (3.5) | 60.0 (12.0) | 13.8 |
| Carbohydrate (g) | 30.0 (12.0) | 129.0 (22.0) | 23.2 | 37.0 (12.0) | 157.0 (29.0) | 23.6 | 40.0 (12.0) | 174.0 (30.0) | 23.0 |
| Total fat (g) | 7.5 (3.0) | 38.0 (7.3) | 19.7 | 7.7 (3.6) | 45.0 (10.0) | 17.1 | 8.1 (3.4) | 49.0 (12.0) | 16.5 |
| Fibre (g) | 2.8 (1.7) | 13.0 (3.6) | 21.5 | 3.7 (1.7) | 16.0 (4.5) | 23.1 | 3.6 (1.7) | 18.0 (4.4) | 20.0 |
| Saturated fat (g) | 3.9 (2.0) | 18.0 (4.9) | 21.7 | 3.9 (2.3) | 20.0 (5.7) | 19.5 | 3.8 (1.9) | 22.0 (6.0) | 17.3 |
| Total sugars (g) | 15.0 (7.5) | 67.0 (16.0) | 22.4 | 19.0 (8.2) | 78.0 (19.0) | 24.4 | 19.0 (7.4) | 83.0 (22.0) | 22.9 |
| Iron (mg) | 1.3 (1.3) | 6.5 (2.0) | 20.0 | 1.4 (1.4) | 7.1 (2.0) | 19.7 | 1.7 (1.5) | 8.1 (2.1) | 21.0 |
| Calcium (mg) | 152.0 (89.0) | 747.0 (193.0) | 20.3 | 210.0 (89.0) | 726.0 (197.0) | 28.9 | 222.0 (92.0) | 762.0 (231.0) | 29.1 |
| Vitamin C (mg) | 6.4 (8.9) | 52.0 (24.0) | 12.3 | 8.6 (13.0) | 69.0 (32.0) | 12.5 | 8.5 (12.0) | 78.0 (40.0) | 10.9 |
| Folate (ug DFE) | 32.0 (46.0) | 254.0 (73.0) | 12.6 | 51.0 (44.0) | 305.0 (94.0) | 16.7 | 50.0 (65.0) | 340.0 (103.0) | 14.7 |
| Potassium (mg) | 380.0 (155.0) | 1801.0 (321.0) | 21.1 | 435.0 (156.0) | 1980.0 (414.0) | 22.0 | 439.0 (165.0) | 2142.0 (466.0) | 20.5 |
| Sodium (mg) | 174.0 (85.0) | 1088.0 (263.0) | 16.0 | 260.0 (94.0) | 1501.0 (369.0) | 17.3 | 265.0 (130.0) | 1713.0 (329.0) | 15.5 |
| Zinc (mg) | 0.8 (0.7) | 6.2 (1.2) | 12.9 | 1.2 (0.8) | 7.1 (1.5) | 16.9 | 1.1 (0.7) | 7.7 (1.7) | 14.3 |

^a^ Usual energy and nutrients intakes calculated by Multiple Source Methods (MSM) over three 24-hour dietary recall
